# Smartphone Technology for Applications in Image-Guided Minimally Invasive Interventional Procedures

**DOI:** 10.1007/s00270-024-03925-4

**Published:** 2024-12-16

**Authors:** Katerina Lee, Pournika Muniyandi, Ming Li, Laetitia Saccenti, Anna Christou, Sheng Xu, Bradford J. Wood

**Affiliations:** 1 Center for Interventional Oncology, NIH Clinical Center and Center for Cancer Research, National Cancer Institute, National Institutes of Health, 10 Center Dr, Bethesda, MD 20892 USA; 2 Hospital of the University of Pennsylvania, Philadelphia, PA USA; 3 University of Missouri-Kansas City School of Medicine, Kansas City, MO USA

**Keywords:** Smartphone technology, Image-guided therapies, Procedural medicine, Virtual reality, Augmented reality, Artificial intelligence

## Abstract

Smartphones contain powerful and enabling technology components that can be re-applied toward procedural tasks commonly used in everyday clinical image-guided therapies and minimally invasive procedures that use three-dimensional information and feedback during procedures. Smartphone applications are a relatively young field of research that have potential in interventional radiology (IR) toward improvement of accuracy and standardization for needle-based procedures like biopsy and ablation. This review explores the adaptation of smartphones’ built-in sensors for virtual and augmented reality uses in IR. The ergonomic advantages and translational hurdles and limitations are reviewed, outlining the potential future developmental pathway of smartphones in IR.

**Supplementary Information:**

The online version contains supplementary material available at 10.1007/s00270-024-03925-4.

## Introduction

The smartphone industry is one of the fastest growing sectors in technology [1]. Since the first appearance of modern smartphones in 1992, rapid technological advancements have led to explosive growth in functionality, speed, and accuracy. Smartphones already play a great role in medical education, diagnostics, and therapeutic decision-making via applications that assist with medical information, research, literature searches, guidelines, algorithms, nomograms, imaging, clinical decision-making, and even procedures.

Smartphones are equipped with numerous sensors and applications that can be adapted or redirected toward medical uses. Smartphone applications for clinical decision-making were recently classified as 33% utilizing a camera, 29% providing guidelines, 18% using sensors, 16% providing predictive modeling, and 4% other categories [2]. Training and education outpace clinical use, with ~ 85% of all Accreditation Council for Graduate Medical Education training program respondents using smartphones, and over half using smartphone applications in clinical practice [3]. Because smartphones are compact, relatively affordable, versatile, and ubiquitous, further expansion, deployment, and validation in the medical field can be expected. In this review, we explore current smartphone applications in procedural and interventional settings, especially image-guided therapies, classified according to the smartphone sensors used and potential future directions of smartphone-assisted procedures. We will discuss the basic components of a smartphone, virtual reality applications, augmented reality applications, and artificial intelligence applications, all with primary focus on applied usage in interventional radiology.

## Components of a Smartphone and Procedural Applications

(Continued in Supplementary Text 1).

The overall design of a smartphone includes the touchscreen display, camera, operating system, network, and many sensors, including an accelerometer, gyroscope, proximity sensor, magnetometer, and others. Smartphones can display images with an expectation of reasonable quality, perhaps suitable for certain standard procedural tasks, like rough guidance of a needle angle, such as for an image-guided biopsy or ablation.

### Camera

The built-in smartphone camera enables a variety of functions beyond imaging, such as face-recognition, document scanning, QR code reading, spatial referencing, or measurements. These features enable built-in smartphone cameras to perform a wide range of clinical tasks, from imaging devices to augmented reality (AR)-guided procedures. For such reasons, smartphone cameras have been applied to procedures and surgeries in many specialties, for procedural planning, navigation, imaging, visualization, composite device placement, and verification (such as with tumor biopsy or thermal ablation). Specific clinical examples of diverse camera applications include percutaneous image-guided biopsy, endoscopy, laryngoscopy, and patient positioning for CT planning. Additionally, smartphone-assisted procedures have been reported in orthopedic surgeries, including total hip arthroplasty [4–6] and knee arthroplasty [7]. In each application, smartphone cameras were used to improve visualization and procedural planning.

### Internet

The internet and available networks are critical enabling factors in communication via smartphones. Smartphone processing units may not have the capacity to rapidly process information quantities above a certain threshold in practical time frames required. In this case, large data may need to be processed or stored remotely on an independent workstation, via internet, cloud, Bluetooth, or network transfer. Smartphone data handling also introduces complex regulatory, security, and privacy issues which may vary depending upon the geography, national laws, research or clinical use, method of use, and intent. Physicians and patients commonly remotely communicate via the internet in telehealth. Diagnostic radiology applications are not reviewed here, but include stroke evaluation via images from non-contrast CT and CTAs uploaded to cloud server and available on smartphones [8].

### Bluetooth

Bluetooth allows short-range communication with proximate external hardware. Pairing yields one-way data transfer, with one device as the receiver, and one as the giver. This enables interfacing with local workstations.

### Audio

Audible feedback can also be used for navigated needle adjustment in percutaneous procedures (e.g., tone change when optimal angle or depth reached or as approaching critical structures) [9]. Audio may also be input for multi-modality artificial intelligence models, where audio acquired with a voice recorder can be converted to mel spectrograms, which model sound frequency over time aligned to a perceptual scale of pitches that humans hears as equidistant, via application of a frequency domain filter, and such images may theoretically be analyzed according to a procedural or medical task, such as determining levels of anesthetic required, analysis of breathing sounds to predict level of awareness or sedation, or classification of procedural pain. Mel spectrograms are used in voice recognition systems such as Siri or Alexa.

### Sensors

Smartphones have many built-in sensors. Motion sensors include accelerometers, gyroscopes, gravity sensors, light detection and range sensors, magnetometers, and rotational vector sensors. Environmental sensors include temperature, pressure, light, and humidity sensors. Position sensors include orientation and magnetic field sensors. Inertial measurement unit (IMU) sensor is typically an integration of gyroscope, accelerometer and magnetometer to output angular velocity, acceleration, and magnetic fields in multiple axes [10]. Base sensors use data from a single physical sensor. In this review, the gyroscope and accelerometer were the most commonly used sensors in procedural applications in image-guided interventions, such as needle-based biopsy and ablation.

**Fig. 1:**
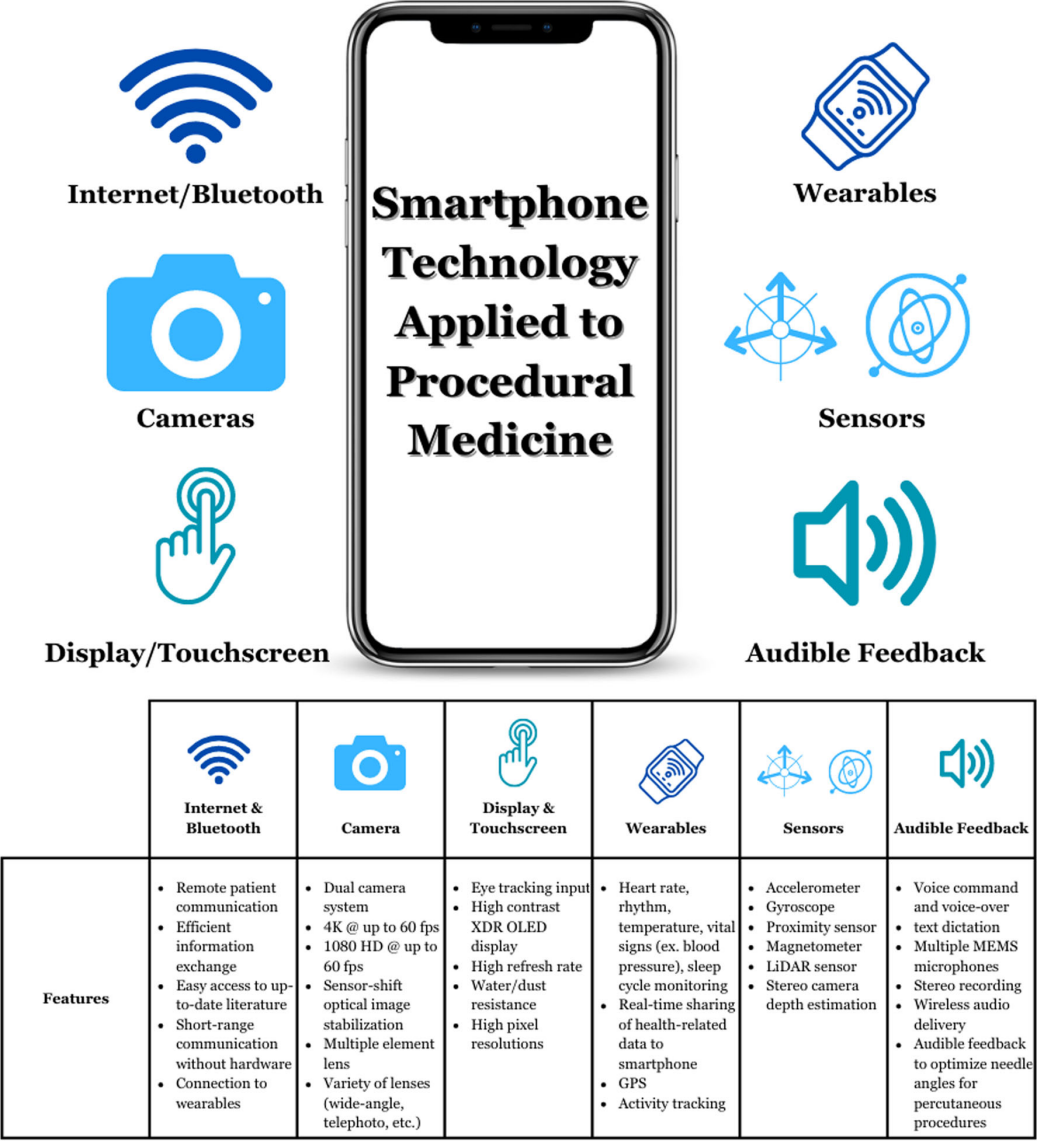
Components of a smartphone and procedural applications. High definition (HD), extreme dynamic range organic light-emitting diode (XDR OLED), Global Positioning System (GPS), light detection and ranging (LiDAR), microelectronic mechanical systems (MEMS)

Accelerometer—The accelerometer measures the acceleration of the smartphone via a Micro-Electro-Mechanical Systems (MEMS)—a system of microscopic mechanical sensors and actuators—that converts the change in speed of a seismic mass into a capacitance change in a circuit [11]. Its role is critical in Global Positioning System (GPS) tracking and measuring the speed of movement (Fig. 1).

**Fig. 2:**
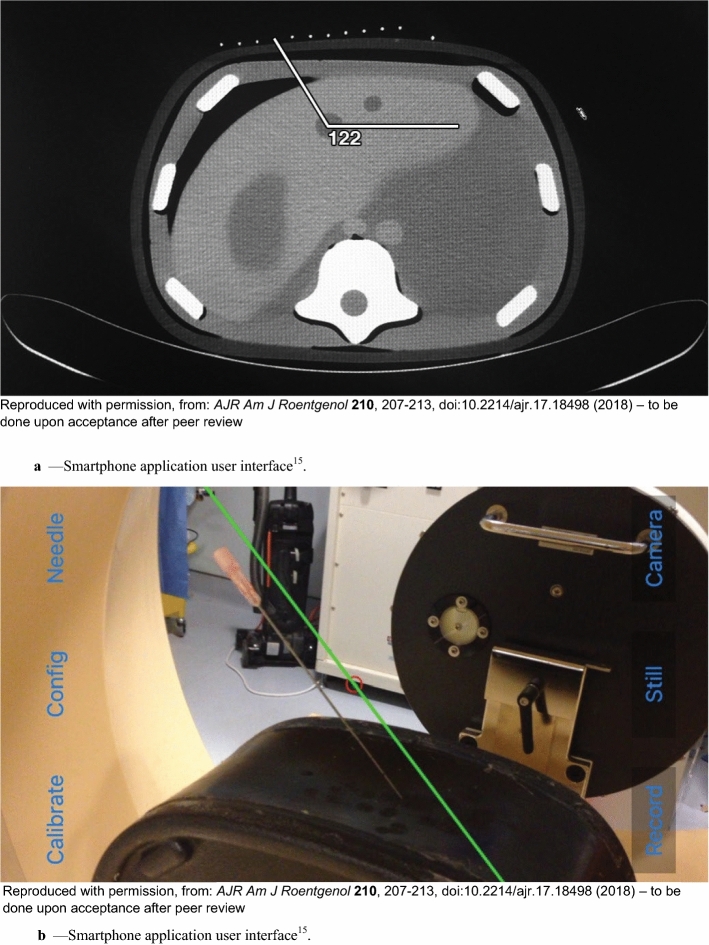
**a** Smartphone application user interface [15]. **a** CT image. Lines show planned path on CT console. Number “122” refers to angle of needle in degrees. **b** Smartphone application user interface [15]. **b** Screen shot shows interface of mobile application. Green line is guideline that shows planned angle on smartphone's screen. Red circle is center of screen. Buttons are shown in blue. Record button is used to start and Still button is used to stop video recording and image capturing. Camera button is used to switch between front and back cameras. Calibrate button is for registering smartphone to CT scanner. Config, abbreviation meaning “Configure,” button is for setting up wireless connection between smartphone and local PC to allow optional image display on PC and additional control from PC. Needle button is used to set planned needle angle on smartphone

**Fig. 3:**
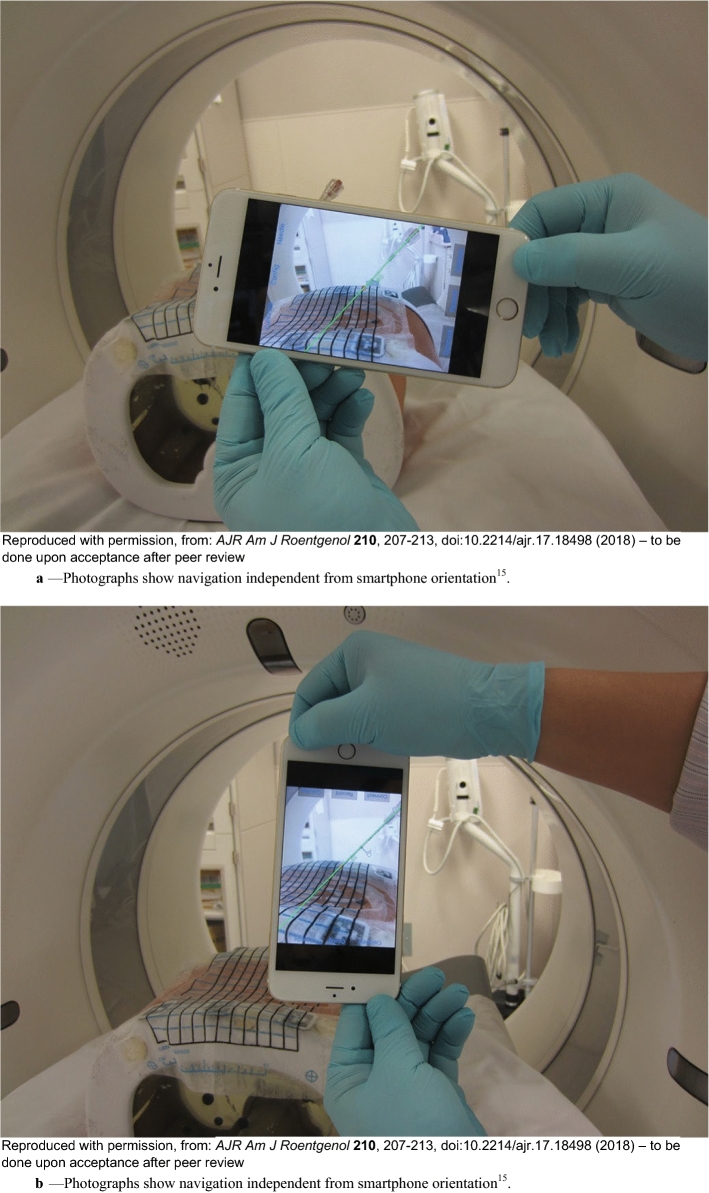
**a** Photographs show navigation independent from smartphone orientation [15]. **a** Photographs obtained with user holding smartphone in landscape **A** and portrait **B** orientations show that path angle (*green line*) displayed on smartphone is always referenced to CT. **b** Photographs show navigation independent from smartphone orientation [15]. **b** Photographs obtained with user holding smartphone in landscape **A** and portrait **B** orientations show that path angle (*green line*) displayed on smartphone is always referenced to CT

**Fig. 4:**
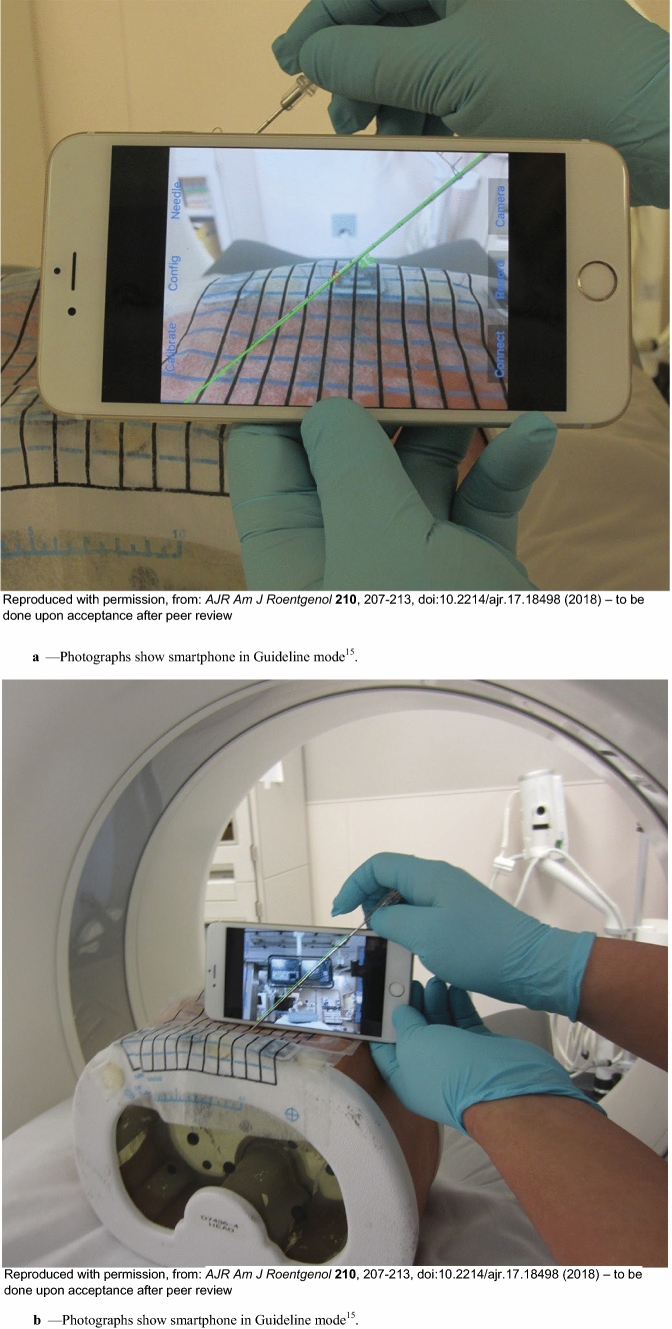
**a** Photographs show smartphone in Guideline mode [15]. **a** Smartphone can be placed either in front of needle **A** or behind needle **B** for guidance. **b** Photographs show smartphone in Guideline mode [15]. **b** Smartphone can be placed either in front of needle **A** or behind needle **B** for guidance

**Fig. 5:**
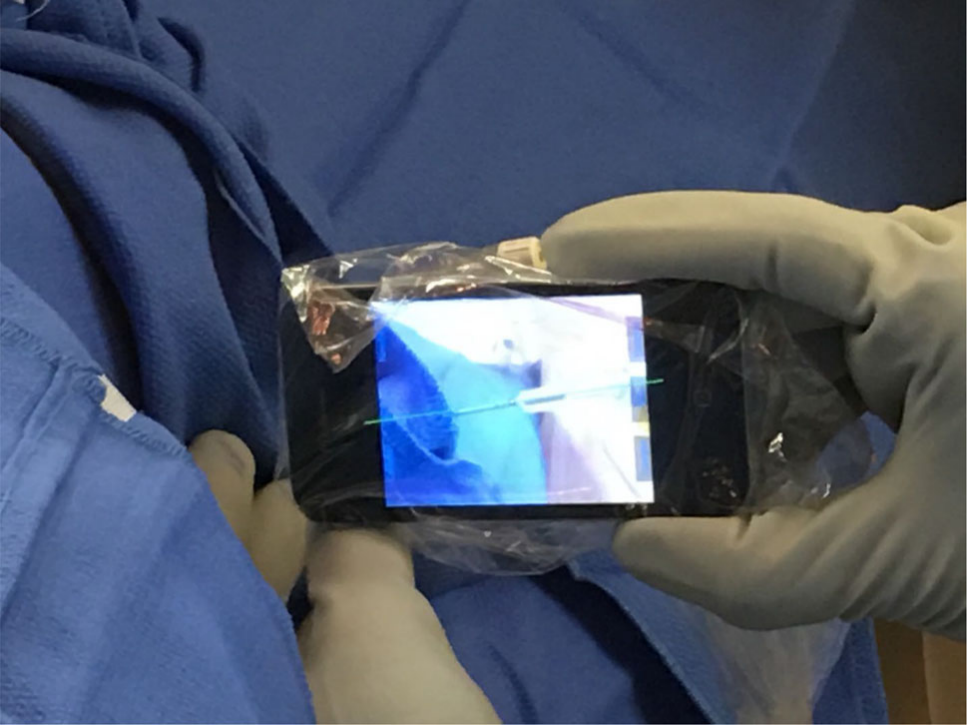
Smartphone with custom application for needle angle selection demonstrated in a real patient biopsy. Smartphone with custom application for imaging the needle and reproducing the angle of superimposed (*green*) needle insertion pathway for a CT-guided biopsy. The pathway was pre-selected with a procedural CT scan and the phone was calibrated to the CT table prior to placement in a sterile translucent bag. The actual needle resides beyond the smartphone, and the shaft and hub are seen superimposed on the needle path plan (*green line*). The application used the smartphone camera and gyroscope, and interfaces with the procedural CT scan DICOM images via Bluetooth

Gyroscope—Like the accelerometer, the gyroscope is also a MEMS. It measures the angular velocity of the smartphone in three axes to determine the smartphone’s orientation. It is often used for navigation systems or smartphone games or photographs that require tilting [12]. Many clinical applications use built-in goniometers, which are a combination of an accelerometer and a gyroscope that can be used to track motion and calculate the orientation of a phone [5, 13, 14]. In addition to being able to analyze motion accurately, a smartphone’s compactness grants operators more freedom of movement for better visualization of targeted anatomy, compared to traditional procedural devices. In fact, a smartphone with a built-in camera and gyroscope was used as an inexpensive, widely available, accurate, and reliable guidance device for CT-guided percutaneous needle-based procedures [15]. A mobile application was developed to facilitate needle angle selection; the application brought the planned needle angle to a real-time, low-cost, intra-procedural display orthogonal to the axis of the needle as well as looking down the axis of the needle (along the shaft and hub). The direct visualization of the planned angle provided an alternative to the heavy reliance on the physician's cognitive visuospatial memory and hand-to-eye estimates and skills (Figs. 2a, b, 3a, b, 4a, b, 5). Combined CT and smartphone guidance was significantly more accurate than CT-only “step-and-shoot” guidance for initial needle angle selection and placement. This led to a smaller final targeting error, suggesting that smartphone guidance can improve the accuracy of needle navigation and placement compared with the conventional method. A recent similar study also highlighted the benefits of a gyroscope in needle puncture with improved accuracy and target hit rate [16].

#### Applications in Interventional Radiology

Smartphone applications in IR are a relatively young space of research. Many studies focus on applications as navigation tools for accurate needle positioning/insertions, as this is a critical step for success in needle-based IR procedures. For this reason, many are exploring the use of virtual and/or augmented reality to bypass larger, bulkier, and more expensive medical devices/technologies to achieve equivalent accuracy and efficiency. Although not many original applications have been published yet, we aim to review the recent exploration of smartphone applications in IR in hopes of stimulating further development of smartphone VR and AR applications intended for procedural settings.

## Virtual Reality Applications

The utilization of VR in the surgical field has experienced rapid growth, such as in teaching demonstrations, training, treatment planning, and surgical navigation [17–19]. The VR market is one of the fastest growing sectors and its global revenue was predicted to surpass $40 billion by the end of 2024, speaking to the potential applications in surgery and image-guided therapies [20]. Certainly, however, funding of business models does not equal evidence of value. VR allows the user to observe and interact with a virtual environment through a system, such as a head-mounted device (HMD) [21]. In surgical training, VR HMDs have been used to create an abstract virtual operation room (OR) for team training scenarios [22, 23]. VR can also simulate real medical instruments, where the computer console generates and maintains force feedback. Currently, most surgical simulations focus on task training of surgical skills, ranging from knot tying to chest-tube insertion to flexible endoscopy [23], though there is room for future expansion. There are also VR systems for teaching anatomy, spatial anatomic relations, or for simulation and planning specific procedures.

Any goggle, wearable, or workstation-based application for IR education or simulation could also be applied via smartphones or smart pads. A smartphone-based approach might carry cost advantages compared to goggle-based VR education, while still maintaining a reduction in factors such as error, time, and radiation exposure [24]. Smartphones may have fewer barriers to adoption for such educational and remote applications, compared to goggles, due to access, ergonomics, calibration requirements, and registration challenges. Additionally, they avoid bulk, sweat, sterility issues, line-of-sight depth cues, cybersickness, accommodation headaches, battery life, or goggle slippage. Currently, IR has fewer VR publications among 10 procedural specialties [25], such as neurosurgery, otolaryngology, vascular, hepatobiliary, orthopedic, plastics, and urology. As such, VR applications in IR remain relatively untouched and exploration should be widely encouraged, lest IR be left with modifying non-IR approaches.

## Augmented Reality Applications

In contrast to VR, AR superimposes digital information onto the real world, creating a technologically-enhanced version of reality that is visualized through either fixed [26], mobile [27, 28], or wearable [29, 30] AR devices. Recently published studies mainly focus on needle positioning/insertions, reporting promising results for this growing need for specific IR procedures. Implementing small and relatively economically sound smartphones for AR platforms may be accurate enough to eliminate the need for expensive and bulky medical imaging devices, although this largely depends on the system tracking accuracy of the device. One common type of tracking is vision-based, which usually occurs by detection of markers or specific features of items being imaged. This vision-based tracking has been shifting from marker-based to simultaneous localization and mapping (SLAM) techniques, where the user location is being actively tracked, while the environment is being mapped simultaneously. This is possible by cameras and inertial sensors, such as accelerometers and gyroscopes that add supplemental information for tracking and orientation. As previously discussed, the processing of this large data can be performed on a remote processor via internet data sharing [31]. The capacity of the processor for rapid intra-procedural processing remains to be fully tested or understood and may depend upon the integration and availability of the processor for specific computationally intense tasks.

**Fig. 6:**
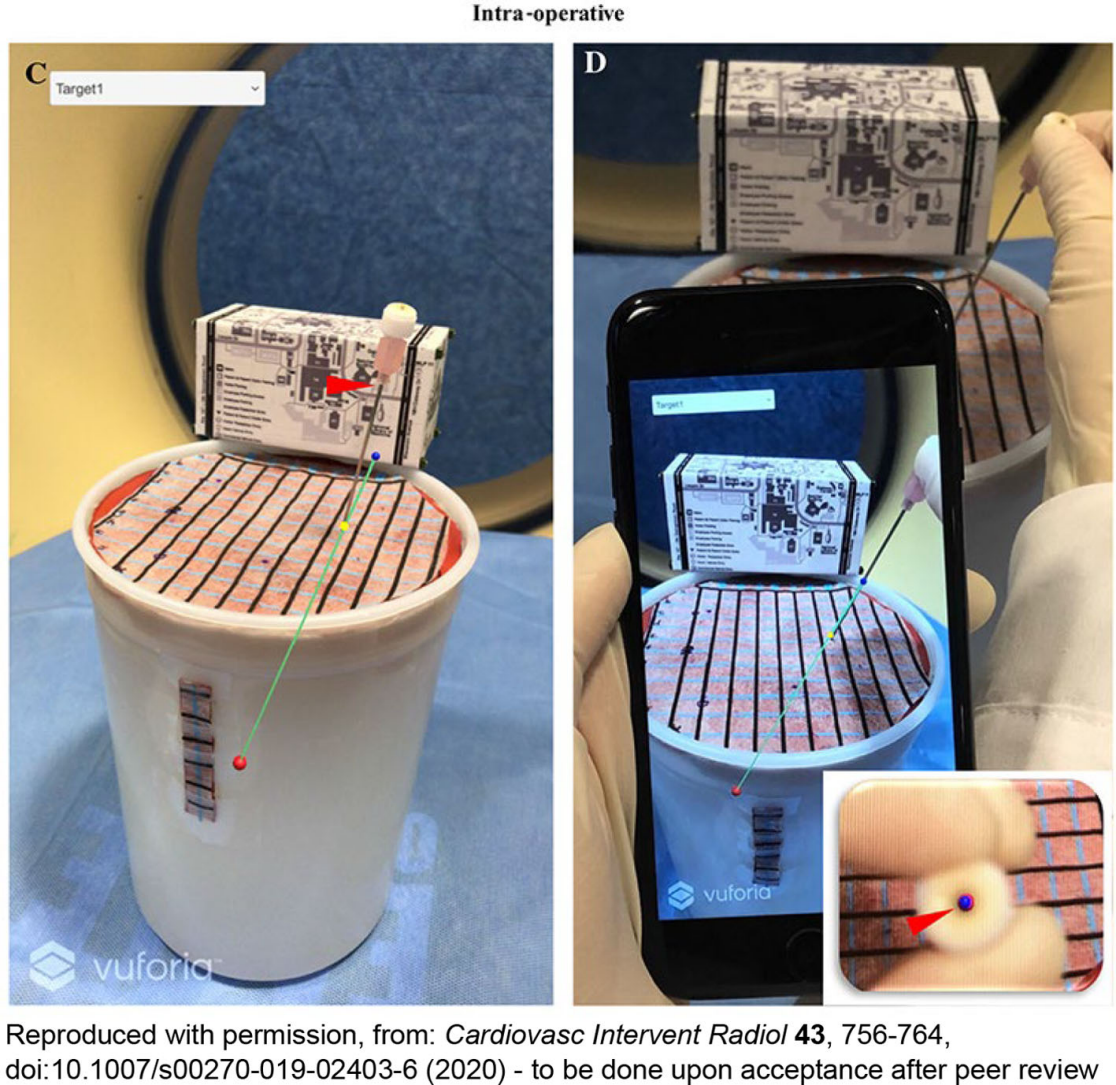
AR Components [32] **C** Smartphone screen with an intentionally off-axis needle placement. A dropdown menu (white box, top left) allows selection of preplanned targets. The smartphone continuously transforms the virtual trajectory relative to the 3D reference marker as the phone is moved and superimposes the trajectory on the image. The AR needle virtual trajectory (*green line*), target (red dot), entry point (yellow dot), and depth marker (navy dot) are components of the smartphone overlay display. When the needle hub base (arrowhead) coincides with the virtual depth marker (navy dot), the needle has been inserted to its proper depth. Note that the yellow dot shown has been added to the image for illustrative purposes (c, d). While the dot was clearly visible within the AR software during use, it was difficult to discern in the screen capture shown. **D**, Smartphone screen displaying a well-aligned needle. The needle is being advanced along its planned AR trajectory, as indicated by the alignment of the needle and AR trajectories. The inset shows a bull’s-eye view, in which the AR needle trajectory, target marker (red dot), and depth marker (navy dot) are all superimposed in the center of the needle hub (arrowhead)

**Fig. 7:**
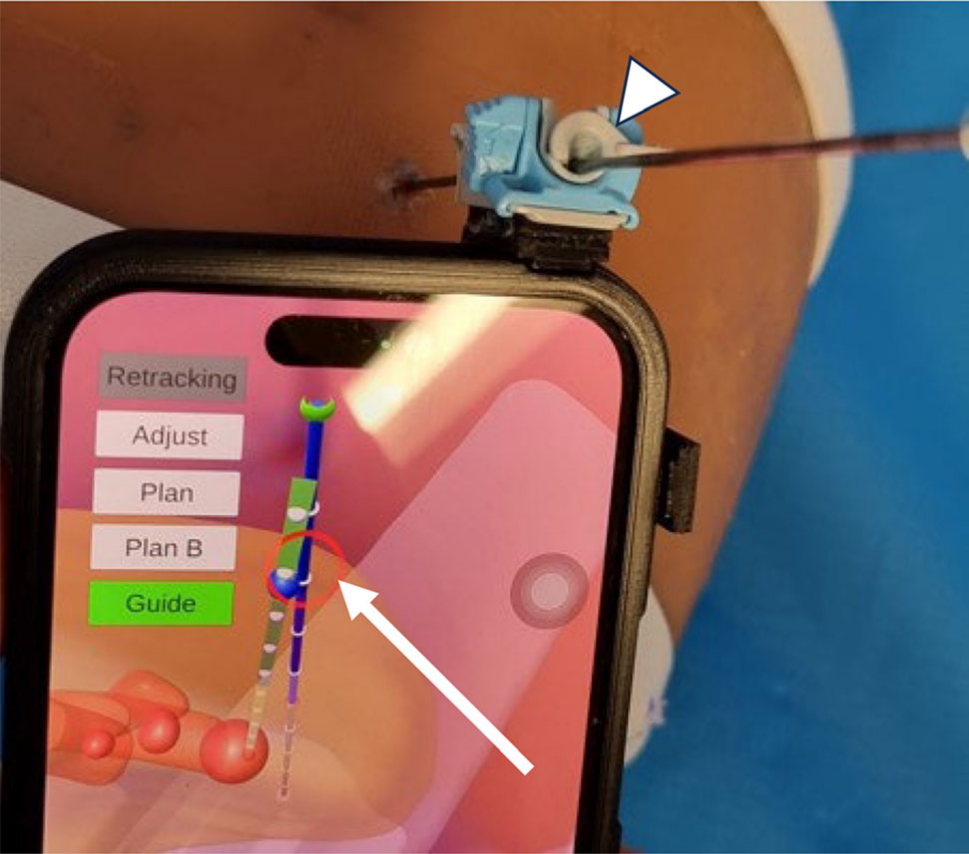
Smartphone AR application with integrated needle guide (arrowhead). Reproduced with permission, from: Scientific presentation at Society of Interventional Radiology (SIR) 2024 Annual Scientific Meeting (Salt Lake City, Utah); abstract is available on JVIR [33]. The current direction of the needle is projected from the attached needle guide as blue line, and the planned trajectory is displayed as green line. When both are aligned, the feedback circle (white arrow) becomes small and green

Several studies have proven the potential benefits of a smartphone-based AR systems during medical procedures. In one study, a smartphone-based AR system was developed to facilitate needle trajectory planning and real-time guidance during percutaneous procedures [32]. The AR software provided interactive functions for image analysis, interventional planning, and registration between the 3D reference marker and the preoperative images, from multiple perspectives. Once planned, the planned and optimized needle trajectories were transferred to the smartphone. The desired initial sequential target was selected (from among the available planned targets) on the smartphone application. The 3D reference marker in the camera’s field of view allowed for continuous, automatic registration and iterative adjustment of the treatment planning, with the smartphone display updated as the smartphone was moved, or needles were placed. This update provided real-time iterative feedback while updating the displayed virtual trajectory and treatment plans accordingly. The preassigned entry point, planned needle trajectory, intended target, and a virtual marker for needle depth were displayed on the handheld smartphone and superimposed onto the physical phantom patient, visible both from side orthogonal and in-line (looking down the needle shaft axis). The operator used the smartphone display to locate the selected skin entry point, align the needle with the planned AR trajectory, and then advance the needle (Fig. 6). The operator thus advanced and adjusted the needle through instantaneous real-time feedback, based on preoperative planning. This utilization of a smartphone-based AR guidance application led to a 78% reduction in the average operator insertion error for AR-guided needles compared to CT-guided freehand, eliminated the need for intraoperative CT scans and radiation, and yielded a 66% reduction in average procedure times [32]. Another smartphone-based AR system has an integrated needle guide into the smartphone cover. The fixed needle guide enables a connection between the real space and the AR space, allowing for new applications including planning the needle trajectory at the patient's bedside (Fig. 7) [33].

**Fig. 8:**
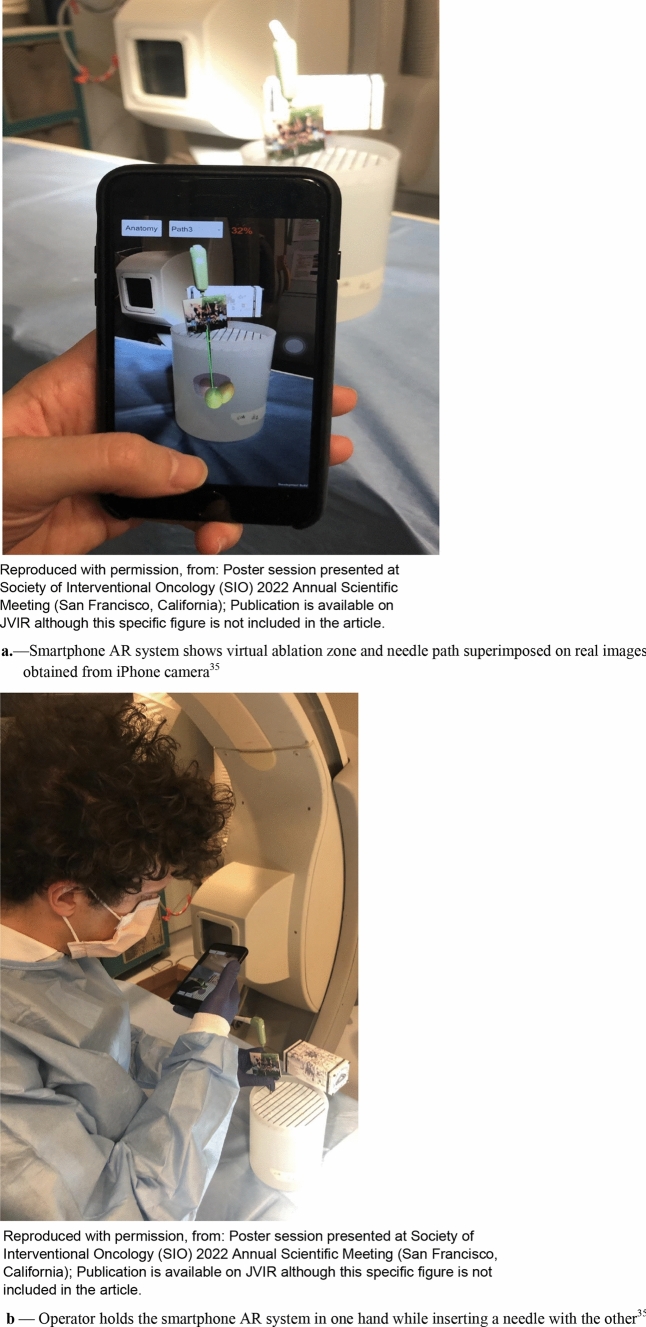
**a** Smartphone AR system shows virtual ablation zone and needle path superimposed on real images obtained from iPhone camera [35], **b** operator holds the smartphone AR system in one hand while inserting a needle with the other. [35]

**Fig. 9:**
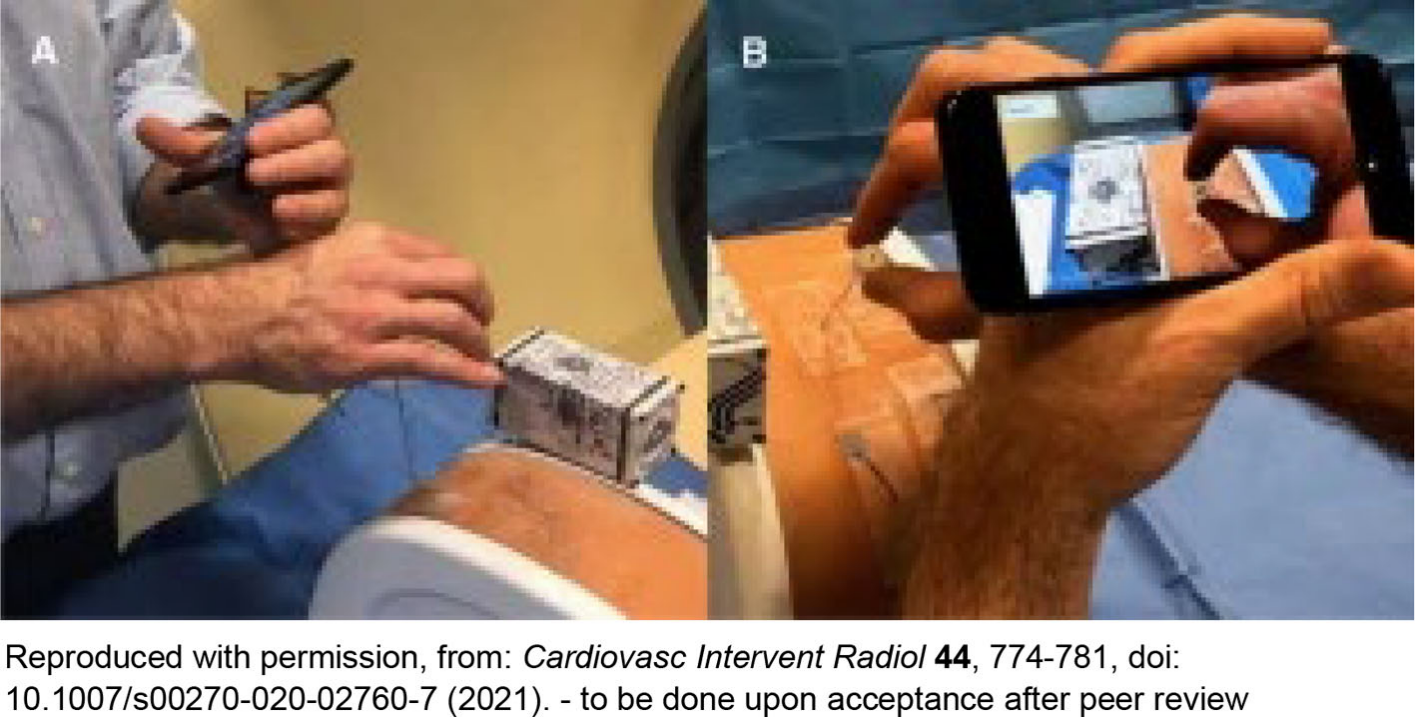
Photographs show how to hold smartphone during interventional procedures [24]. Smartphone may be hand-held with one hand stabilizing the phone and other hand redirecting the needle

Smartphone AR was also compared to smartglasses-based AR in terms of system accuracy and needle placement performance for percutaneous needle interventions on a 3D-printed phantom [34]. The target overlay error and the needle overlay angle error of the smartphone were comparable to HoloLens. However, the smartphone-guided needle placements showed reduced error compared to the HoloLens1 but slightly increased time (87 ± 17 s, 71 ± 27 s, respectively, *p* = 0.02) [34]. Both AR devices reduced placement error compared to conventional CT-guided freehand. Both studies effectively demonstrated the potential of smartphone-based AR platforms toward guiding percutaneous biopsies and ablations. Additionally, smartphone AR had less residual tumor and more efficiency than free-hand ablation tasks for multi-probe composite ablations [35] (Figs. 8a, b). Benefits of smartphone AR included improved needle insertion accuracy, reduced procedural times, and decreased radiation exposures. With a smartphone AR, these benefits come without the cost, ergonomics, accessibility, headaches and goggle sickness, or other challenges of goggle-based AR. Note, AR-enabled smartphones or smartpads can be stabilized manually or with a mechanical arm. Either way, sterility issues might be worked out with a wipe down and a sterile translucent bag (like a patient consent pad or ultrasound probe cover) [24] (Fig. 9). Perhaps most importantly, smartphone AR allowed inexperienced operators to perform similar to expert operators, supporting standardization of IR procedures, which might allow enhanced reproducibility and reliability for IR procedures, and shorter learning curves.

Table 1 lists smartphone applications that use AR systems in IR. All these devices use cameras and multiple motion sensors to serve as a navigation system. The most common benefit is that AR systems allow real-time data extraction and analysis, which translates to more accurate and interactive procedures by the operators. This is especially helpful in procedures that require real-time tracking and needle navigation such as for biopsy and ablation [24]. Procedure time and radiation were reduced when placing needles using cone-beam computed tomography (CBCT)-guided fluoroscopy, AR guidance on smartphone, and AR guidance on smart goggles, comparing to conventional free-hand cognitive estimates. A significant reduction in needle placement time for both AR platforms was seen, while eliminating radiation exposure.

**Table 1:** Review of interventional radiology smartphone applications including the main smartphone sensors they use and their benefits

Author	Type of study	What it was used for	Usage	Benefits
Hirata et al. [41]	Phantom experiment	Needle insertion	Smart Puncture/Smart Puncture Line displays predetermined angle of insertion and maintains a fixed angle with respect to gravity	- Procedure can be performed without an assistant - No cleaning required, and smartphones are portable - No additional equipment or installation beyond smartphone required - Conventional (non-CT fluoroscopy-guided) punctures do not provide real-time tracking of the needle, so physicians have to advance the needle without a reliable guide. This technique could hopefully help puncture accuracy and decrease imaging frequency
Zhao et al. [42]	Phantom experiment	Needle tracking/insertion	Smartphone’s magnetometer detects the magnetic field from the permanent magnet (signal emitter) and calculates the relative position information based on an algorithm	- Avoids the drawbacks of optical tracking, such as needing a line-of-sight which makes tracking internal targets difficult, and of EM tracking (high costs and complex procedures) - Standard needles do not need to be modified
Long et al. [24]	Phantom experiment	Needle tracking/insertion	Smartphone AR application displays superimposed virtual path, target, entry point, and final position of the proximal end of the needle and bull’s-eye view along the axis of the trajectory guides the initial puncture	- Automatic registration and real-time superimposing of needle trajectory on phantom image on smartphone screen - Accurate needle placement, while reducing time, radiation exposure, ergonomics, and inter-user variability compared to CBCT-guided fluoroscopy
Xu et al. [15]	Phantom experiment	Needle angle selection and guidance	A developed smartphone application was used to reproduce the planned needle angle as the actual insertion angle via real-time intraoperative display. The guideline in the display was compared to the actual needle prior to angle selection	- Low-cost and widely available hardware Method is accurate, effective, and easy to implement - Reduces reliance on operator’s visuospatial ability to estimate and reproduce angles - Allows for use of standard needles without additional disposable cost to procedure like electromagnetic or optical tracking-based navigation systems
Hecht et al. [32]	Phantom experiment	Needle trajectory planning and real-time guidance	A developed AR guidance smartphone application used CT imaging data and a 3D reference marker to identify targets and entry points for needle insertions. Camera settings were used to align needle with planned AR trajectory, advance the needle, and adjust based on real-time feedback	- Improved needle delivery accuracy compared to conventional CT-guided freehand navigation → potential for improved ablation margins - Eliminates need for intraoperative CT scans - Reduces procedural time - Platform for physician training and standardization - Cost-effective technology - Ergonomic advantages of using smartphone rather than googles/head-mounted displays (decreased eye fatigue, system lag, etc.)
Li et al. [34]	Phantom experiment	Needle placement guidance	A developed AR platform superimposed annotated anatomy and a planned needle trajectory onto patient in real-time. The platform was employed on a smartphone and smartglasses	- AR smartphone displays large field of view of environment and the region of interest compared to smartglasses -Visualization can be quickly shared and experienced by multiple users which allows intraprocedural feedback - Simple and cost-effective - Minimal added time for operator training and preprocedural planning - Can be used for training less experienced operators - Marked improvement in needle placement accuracy - Accurate image and needle overlay for object visualization and needle guidance
Morita et al. [43]	Phantom experiment	Needle placement guidance	An AR application that displays protractor registered to anatomy to guide needle placement and insertion	- Accurate needle placements - Less radiation exposure - Smartphone does not need to be in contact with the needle
Sanchez et al. [44]	Experiment	Display monitor for dosimetry records	An application to display personal dosimeter doses during fluoroscopy-guided procedure	- Safety measure of cumulative dosimetry exposure - Measure to improve radiation protection
Saccenti et al.^33^	Phantom experiment	Needle trajectory planning and real-time guidance	A developed AR guidance application with an integrated needle guide on the smartphone cover. Camera, gyroscope and magnetometer are used for real-time feedback during needle placement	- Improved needle delivery accuracy compared to freehand navigation - Needle trajectory can be planned at patient’s bedside - No difference of accuracy according to operator’s experience - The operator can hold both the smartphone and the needle with one hand

Radiation exposure should always be minimized to as low as reasonably achievable in procedures [7, 24]. Another interesting feature of any AR platform is perhaps enhanced concentration or focus, via eliminating the need to look back and forth between the control room, monitor, and the patient. During craniotomy planning, use of an AR system reduced the number of attention shifts in physicians, while delineating depicting a tumor superimposed upon the skull [36].

While the potential for AR and smartphone applications in procedural settings is vast, there are limitations to this approach. AR systems heavily rely upon accurate registration and tracking systems of human anatomy and procedural instruments. A recent study implemented rigid fixture of needle to smartphone, which could be another way to improve accurate tracking, requiring less reliance on registering fiducial markers [37]. While rigid structures have been proven highly successful with registration in surgery or procedures in bone, brain, or prostate, dynamic moving structures due to either functional movement, organ shift, or from translation of ventilation are difficult to register and track [38], opening up another fertile ground for future exploration.

## Smartphone Artificial Intelligence

Similar to AR, mobile platforms have been used to bidirectionally both train and apply artificial intelligence (AI) in medicine. Generalizable AI models and algorithms may be implemented on mobile devices for a variety of applications, such as classification into disease categories. In some cases, smartphones are used to collect information from users to train AI algorithms that might also be subsequently deployed on smartphones. For example, for dermatology purposes, smartphone images of faces were used to train a model to detect and grade acne (Global Acne Severity Scale), which performed with weighted average of precision of 84% for inflammatory lesions, 61% for non-inflammatory lesions, and 72% for post-inflammatory hyperpigmentation [39]. A smartphone AI system was trained to detect diabetic retinopathy from direct imaging of patient eyes with smartphone cameras with a sensitivity of over 95% [40]. Because smartphones are ubiquitous, large data required for deep learning may be rapidly captured, shared, and curated. Whether in the format of images, audio spectrograms, sleep activity, vital signs, or peripherally acquired data from wearables, mobile platforms offer a myriad of possibilities toward the training, design, and implementation of AI algorithms. AI-enabled smartphones may soon semi-automatically link to suggested relevant content on PubMed or elsewhere with image or audio input from a procedure or surgery.

Numerous limitations constrain expedited deployment of smartphones in clinic. Hurdles and challenges to adoption and implementation of smartphones in healthcare include hesitation or unfamiliarity with the capabilities, system inaccuracies, variabilities in use, device error, as well as regulatory, licensing, privacy, and data transfer limitations. There remain few published studies with most data being pre-clinical in immobile phantoms without respiratory motion. Licensing, intellectual property challenges, and unconventional business models may also be hurdles to translation of smartphone applications from bench to clinic. Smartphone stabilizing arms and hardware are also not usually manufactured to medical level of accuracy and precision. Some operators however may still ergonomically prefer holding a smartphone over wearing goggles, which may outweigh the limitations.

Although this review focused on procedural and image-guided therapy applications, such as needle-based interventional radiology procedures like biopsy and ablation, this represents a small fraction of possible educational, diagnostic, and patient monitoring applications. As wearable and smartphone technology evolves, unmet clinical needs may be approached via smartphone augmentation of device navigation, education, or access to information.

## Conclusion

Smartphone component technologies may be applied to image-guided procedures to address unmet clinical needs or augment or standardize existing practices. Smartphones hold the potential to impact standardization of manual procedures otherwise dependent upon operator’s cognitive estimation of tasks requiring well-developed hand–eye coordination. The impact of smartphone AI, AR, and VR upon training programs and needle-based procedures remains speculative, but merits attention, since any IR application for goggle-based AR or VR is adaptable to smartphones. Smartphone AR can provide needle guidance accuracy in pre-clinical models that is similar to other navigation technologies in clinic. Hurdles and challenges to adoption and implementation of smartphones in healthcare include hesitation or unfamiliarity with the capabilities, systematic flaws, user variabilities, as well as regulatory, privacy, and data transfer limitations. Future research may focus on real-world accuracy in clinic, hardware, and software integrations and needle guides, as well as adaptation to specific business models and workflows.

It has been established that dataflow and procedural workflows may be facilitated or standardized via smartphone technologies gathering, presenting, or using information during procedures. Smartphone (and thus smartpad) technologies are accessible, low-cost, and accurate for IR procedures requiring needle placement and merit further investigation of hurdles to clinical deployment.

## Supplementary Information

Below is the link to the electronic supplementary material.

Supplementary file1 (DOCX 24 kb)
