# Supplementary material for: Smartphone Technology for Applications in Image-Guided Minimally Invasive Interventional Procedures
Source: Cardiovasc Intervent Radiol. 2024 Dec 16;48(2):142–56. doi: 10.1007/s00270-024-03925-4 (PMC11790737; doi:10.1007/s00270-024-03925-4)
Supplement: Supplementary file 1 — Supplementary file1 (DOCX 24 kb) [file 270_2024_3925_MOESM1_ESM.docx]

**Title: Smartphone Technology for Applications and Image Guided Minimally Invasive Interventional Procedures**

**Journal:** CardioVascular and Interventional Radiology

# **Authors:** Katerina Lee^1, 2^, Pournika Muniyandi^1,3^, Ming Li^1^, Laetitia Saccenti^1^, Anna Christou^1^, Sheng Xu^1*^, Bradford J. Wood^1*^

^1^Center for Interventional Oncology, NIH Clinical Center and Center for Cancer Research, National Cancer Institute, National Institutes of Health, Bethesda, Maryland

^2^Hospital of the University of Pennsylvania, Philadelphia, Pennsylvania

^3^University of Missouri-Kansas City School of Medicine, Kansas City, Missouri

*Authors contributed equally

**Corresponding Author:**

Bradford J. Wood

[bwood@cc.nih.gov](mailto:bwood@cc.nih.gov) | (301) 443-8191 or (301) 728-0362

Center for Interventional Oncology, National Institutes of Health, 10 Center Dr, Bethesda, MD 20892

**Supplemental Materials**

**Supplementary Text 1. Components of a Smartphone and Procedural Applications**

The basic components and established features (e.g., single front and rear camera, GPS, activity tracking, orientation activated commands, multiple speakers/microphones for audio capture and playback, etc.) are commonly present even in entry-level smartphones [5] (Figure 1). Other emerging features (e.g., millimeter wave radar for gesture commands, dedicated processors for deep learning, optical image stabilization, stereo camera depth estimation, voice activated interface control, etc.) may be available on high-end smartphones, but are not ubiquitous across all smartphones [5].

*Display*

Many features of a smartphone display are geared towards creating an intuitive user interface. Instead of having to manipulate a mouse or a cursor on the monitor, the touchscreen allows the user to directly interact with visuals in the display. In a procedural setting, eliminating an extra device to handle, such as a mouse, should simplify and save time. Eye tracking input is expected to expand and may simplify ergonomics. Most smartphones also have high resolution for accurate display of fine details. An iPhone 14 (Apple, Inc.) comes in either a 6.1-inch (diagonal) or 6.7-inch (diagonal) Super Retina extreme dynamic range (XDR) organic light-emitting diode (OLED) display with 2532 by 1170-pixel resolution at 460 pixels per inch (ppi) or 2778 by 1284-pixel resolution at 458 ppi, respectively, with refresh rate of 60Hz [6]. 1080-pixel resolution is the standard for high definition (HD) display [7].

*Camera*

An iPhone 14 (Apple, Inc.) has a dual camera system with 12 megapixels (MP) main (26 mm, ƒ/1.5 aperture, sensor‑shift optical image stabilization, seven‑element lens, 100% Focus Pixels) and 12 MP ultra-wide (3 mm, ƒ/2.4 aperture and 120° field of view, five‑element lens). It also allows video recording at 4K video recording at 24 fps, 25 fps, 30 fps, or 60 fps and 1080 HD at 25 fps, 30 fps, or 60 fps [6]. To put this in perspective, standard movies and television shows are captured at 24 fps, which is the minimum speed for perception of realistic motion [8]. Recent smartphones have multiple cameras embedded with different types of lenses including wide-angle, ultrawide, telephoto, monochrome sensor, or time-of-flight camera (depth sensor). These lenses have different focal lengths, enabling smartphones to capture images with a wider depth of field, and better-quality images with less requisite light.

*Internet*

A myriad of apps facilitate or provide platforms for information exchange with or without direct access to an electronic health record. For example, the Doximity app (Doximity Inc, San Mateo, CA, USA) enables licensed physicians to register, share and discuss Health Insurance Portability and Accountability Act (HIPPA)-protected patient information for clinical decision making [1]. The most ubiquitous medical use of smartphones by providers is likely searching the internet for clinical guidelines or peer-reviewed literature to access the latest updated information and data.

*Bluetooth*

When the receiver device is discoverable, the giver will request to pair, and when the request is accepted by the receiver device, the two will be linked. Then, the transmitter can send images, videos, and other data rapidly via Bluetooth. Upon completion of the transmission, the two devices will disconnect, but remain memory-bonded, so that they can automatically reconnect for sharing data again in the future. Numerous “wearables” transmit health-related data via Bluetooth (smart watches, Fitbit, Kardia, Oura). Wearables (such as Oura rings, Oura, Finland) can continuously monitor users’ heart rates and rhythms, temperature, vital signs, activity, and sleep cycles. This data is shared in real-time to smartphones via Bluetooth to track daily activities, vital signs, and health information. While Bluetooth permits easy sharing of data, one limitation is that the two devices must be within a threshold proximity of each other, and security vulnerabilities differ among transfer between network, Bluetooth, cloud, and the internet.

*Audio*

One of the most overlooked aspects of smartphones is sound quality. Many sources of smartphone entertainment and communication are audio-driven. Voice command and voice-over features improve accessibility for impaired users, allow for no-contact use, and enable dictating text or distinguishing icons via verbal feedback [13]. In addition to the standard microphone, smartphones can have 3-4 microelectromechanical system (MEMS) microphones that require minimal functional power and take up little space [14]. These microphones use electret, a permanently charged material that eliminates the need for a conventional polarizing power supply. Additional microphones can deliver stereo recording, zoom in on desired sounds, or cancel background noise when desirable.

The audio experience has also transitioned from traditional wired “in and out” headphones to wireless audio delivered via Bluetooth. However, this adds the challenge of maintaining audio quality over a wireless link due to an outdated default codec initially chosen for Bluetooth. A codec breaks up audio streams into sizable chunks, compresses the data, then manages the process of sending the data to ensure maintenance of consistent signal integrity [16]. However, the current low complexity sub-band codec (SBC) used in Bluetooth has limitations with bandwidth, processing, and power requirements. New codecs (i.e., Qualcomm’s aptX Adaptive) are able to support higher transfer rates and thus improved audio quality with reduced latency. Ultimately, building flexible codecs with improved data transfer abilities should allow for future enhancements such as multi-channel audio simulation, surround sound, and directional audio for virtual/augmented reality.

*Sensors*

Proximity sensor – The proximity sensor detects the proximity of the user by reflecting infrared light. Such a feature allows the phone screen to turn off during phone calls to prevent the user from unintentionally pressing buttons and to save battery. Proximity sensors are also commonly used for monitoring daily activity and falls [19].

Magnetometer – The magnetometer measures the magnetic field to differentiate north from south. Like the accelerometer, it plays a crucial role in GPS and movement tracking. Smartphone magnetometers enable and inform contact tracing of infectious diseases such as influenzae or COVID-19, via alert and identification of two phones (individuals) who were in close proximity [25]. Smartphone needle tracking devices that used the magnetometer are able to successfully track needles, while avoiding the need for a “line-of-sight”, as required for optical and electromagnetic tracking [26].

LiDAR (light detection and ranging) sensor – LiDAR sensors work by emitting pulsed laser and detecting the time for light return, after bouncing off surrounding environment. Millions of these light pulses replicate the three-dimensional environment precisely [27]. Apple (Apple, Inc.) integrated LiDAR sensors in its recent devices which enable iPhones to start AR apps more quickly and scan and build a more complicated surrounding environment. This is similar to how AR headsets, such as HoloLens, analyze the surroundings and rebuild it virtually. Similar technologies have been used for surgical navigation and visualization, via optically-tracked laser scanning and registration of organ surfaces during surgery [28].

**Supplementary Text 2. Platforms & Regulatory**

*Platforms*

Applications for smartphones are developed using different platforms depending on the type of smartphone and its operating system (OS). The two most widely used operating systems are iOS (iPhone Operating System) by Apple Inc. and Android by the Open Handset Alliance led by Google Inc. For each operating system, programming platforms called Integrated Development Environment (IDE) are used to develop a variety of applications. For example, Xcode (Apple Inc.) for iOS and Android Studio (Google, Inc) for Android. There are also cross-platform IDEs, including Unity (Unity Software Inc.), from which users can create apps useable for multiple operating systems. This allows developers to target a bigger audience with a wider range of devices and platforms. The performance stability, privacy, security needs to be well considered for best practices during mobile application development. It includes security of data storage, permission, networking, communicating, etc. These practices not only ensure user trust and device integrity, but also meet the FDA regulation for medical device.

*Regulatory*

While smartphone medical applications have many benefits and are rapidly evolving, rapid deployment of such technology also carries risks, such as security threats to the highly sensitive private health data. Smartphone security is somewhat dependent on the user’s timely OS updates, which cannot be monitored or enforced. When security is compromised, smartphones may be hacked and patient health information may be at risk for portals or breaches of confidentiality [29]. In procedural applications, there is also the need to constantly ensure that applications are stable and functioning accurately, since technological glitches or code bugs in procedural settings may have detrimental implications. For such reasons, any applications developed for medical usage should be continuously monitored and reviewed meticulously, and are regulated carefully, depending upon the indications for use.

As with any medical device, smartphone medical applications are regulated for safety, effectiveness, and security. Per U.S. Food and Drug Administration (FDA), software functions can be categorized either into those that are of enforcement discretion or at the focus of regulatory oversight. The latter group includes 1) functions that control other devices or analyze medical device data by acting as an extension of another medical device, 2) those that allow mobile platforms to function as medical devices via built-in features/sensors of the platform, and 3) those that analyze patient-specific information to provide diagnosis and/or treatments [30]. Depending on the intended use, indications for use, and risks, these regulated devices are classified into class I: general controls, Class II: General controls and special controls, and Class III: general controls and premarket approval, each with its own criteria. Class II and III devices are evaluated for safety and effectiveness prior to public market. General requirements for these regulated software functions include establishment registration and medical device listing, investigation device exemption (IDE) requirements, labeling requirements, premarket submission for approval or clearance, quality system regulation, medical device reporting (MDR) for adverse events, and correcting problems. The Federal Trade Commission (FTC) released an online interactive tool which helps developers determine which laws and regulations their devices must comply by, including HIPAA for security of patient information, Federal Food, Drug, and Cosmetic Act (FD&C Act) for safety and effectiveness, and Federal Trade Commission Act (FTC Act) for prohibiting deceptive or unfair acts [31]. The landscape for approvals may also depend upon context of use, such as OHSRP/IRB/ or research Board approvals for experimental or research or off-label use of approved device toward exploratory goals.

Despite regulations, smartphone medical applications continue to be developed because of their accessibility and ease of implementation in many integrated medical settings. Of the many applications, recent developments have led to smartphones implementing virtual reality (VR), AR, and artificial intelligence (AI). As patients and providers become more familiar with telehealth, virtual examinations, and smartphone functionality, the breadth of use and indications should increase, but should not outpace rigorous validation of safety and efficacy with high level of evidence and proven value.
